# A Subunit of ESCRT-III, MoIst1, Is Involved in Fungal Development, Pathogenicity, and Autophagy in *Magnaporthe oryzae*

**DOI:** 10.3389/fpls.2022.845139

**Published:** 2022-04-07

**Authors:** Lixiao Sun, Hui Qian, Minghua Wu, Wenhui Zhao, Mengyu Liu, Yunyun Wei, Xueming Zhu, Lin Li, Jianping Lu, Fucheng Lin, Xiaohong Liu

**Affiliations:** ^1^State Key Laboratory for Managing Biotic and Chemical Treats to the Quality and Safety of Agro-Products, Institute of Biotechnology, Zhejiang University, Hangzhou, China; ^2^State Key Laboratory for Managing Biotic and Chemical Treats to the Quality and Safety of Agro-Products, Institute of Plant Protection and Microbiology, Zhejiang Academy of Agricultural Sciences, Hangzhou, China; ^3^College of Life Sciences, Zhejiang University, Hangzhou, China

**Keywords:** *Magnaporthe oryzae*, MoIst1, fungal development, pathogenesis, autophagy

## Abstract

The culprit of rice blast, *Magnaporthe oryzae*, is a filamentous fungus that seriously affects the yield and quality of rice worldwide. MoIst1, a subunit of ESCRT-III, is involved in identified ubiquitinated proteins and transports them into the intraluminal vesicles of multivesicular bodies (MVBs) for degradation in lysosomes. Here, we identify and characterize MoIst1 in *M. oryzae*. Disruption of MoIst1 leads to a significant decrease in sporulation and formation of appressoria, defects in response to oxidative stress, cell wall stress, hyperosmotic stress, and reduced pathogenicity. Deletion of MoIst1 also caused the decreased Pmk1 phosphorylation levels, appressorium formation, the delayed translocation and degradation of lipid droplets and glycogen, resulting in a decreased appressorium turgor. In addition, deletion of MoIst1 leads to an abnormal autophagy. In summary, our results indicate that MoIst1 is involved in sporulation, appressorium development, plant penetration, pathogenicity, and autophagy in *M. oryzae*.

## Introduction

The culprit of rice blast, *Magnaporthe oryzae*, a filamentous fungus, has been used as one of the model fungi for studying the interaction between pathogens and host plants because of its typical growth, development and infection mechanism. Appressoria produced by the conidial germination of *M. oryzae* are a powerful weapon that can destroy the leaf epidermis with a turgor of up to 8.0 MPa (Fernandez and Orth, 2018). The turgor of up to 8.0 MPa within the appressorium originates from the glycerol produced by the degradation of glycogen and lipid droplets transported from the conidia to the appressoria (deJong et al., 1997; Thines et al., 2000). The penetration peg produced under the pressure of huge turgor penetrates the leaf epidermis, and then the primary hyphae extend in the cells to form secondary infection hyphae that span the cells and leads to the appearance of necrotic lesions that can produce abundant conidiophores and conidia, beginning a new cycle (Kankanala et al., 2007).

The endosomal sorting complexs required for transport (ESCRT) system required for transport not only affects the degradation of ubiquitinated membrane proteins, but also participates in the formation of MVB (Katzmann et al., 2001; Babst et al., 2002a,b; Hanson and Cashikar, 2012). ESCRT-0, -I, -II, -III, and several accessory proteins together constitute ESCRT (Hurley, 2008, 2015). Previous studies have shown that ESCRT-III contains four core subunits (Vps20, Snf7, Vps24, and Vps2) and three accessory components (Did2, Vps60, and Ist1) (Schmidt and Teis, 2012). The seven subunits are recruited in an orderly manner to the endosomal membrane and assemble into a complex to function (Babst et al., 2002a). In this research, we mainly focused on MoIst1, a subunit of ESCRT-III. In *Arabidopsis thaliana*, deletion of ISTL1 caused lethality to partial pollen and also played a part in the early endosomal pathway and exocytosis (Goodman et al., 2021). A report on humans showed that IST1, as a part of ESCRT, also worked similarly to other ESCRT components and was involved in HeLa cell cytokinesis (Bajorek et al., 2009).

In addition to the abovementioned functions, ESCRT has also been shown to be involved in autophagy. The ESCRT mechanism mediates autophagy mainly by participating in two of several important stages of autophagy: phagophore closure and autophagosome fusion with lysosomes (Hurley and Hanson, 2010). After discovering that the Vps21/RAB5 GTPase module is involved in phagophore closure in yeast (Zhou et al., 2019b), the authors discovered its specific regulatory mechanism in recent studies; that is, Vps21 regulates the recruitment of ESCRT to phagophores by controlling the interaction of Atg17-Snf7 to catalyze the closure of autophagosomes (Zhou et al., 2019a). Studies in *Arabidopsis* have found that in *chmp1* (a paralog of Did2, also a subunit of ESCRT-III), autophagosomes fail to close normally, thus hindering autophagy (Spitzer et al., 2015). Studies in *Drosophila larvae* have also shown that deletion of ESCRT-I, -II or -III proteins will lead to an increase in the number of autophagosomes (Rusten et al., 2007). In cortical neurons and flies, the deletion of mSnf7-2 or CHMP2B^Intron5^ expression (both of which are subunits of ESCRT-III) will also result in an increase in the number of autophagosomes (Lee et al., 2007). In summary, the absence or dysfunction of ESCRT-III affects phagophore closure and the fusion of autophagosomes with lysosomes, resulting in abnormal autophagy (Lee et al., 2007; Rusten et al., 2007). In addition to autophagy, recent studies in yeast have proven that ESCRT is also involved in the regulation of endoplasmic reticulum autophagy (ER-phagy) and that deletion of ESCRT reduces or blocks ER-phagy (Schafer et al., 2020).

Although the importance of IST1 has been proven in different species, its function in filamentous fungi has not been studied to the best of our knowledge. In this research, we identified that MoIst1, a subunit of ESCRT-III, is involved in sporulation, appressorium development, plant penetration, pathogenicity, and autophagy in *M. oryzae*. Deletion of MoIst1 led to a decrease in sporulation and appressorium turgor, defects in response to oxidative stress, cell wall stress, hyperosmotic stress, translocation and degradation of lipid droplets, and glycogen and reduced pathogenicity. Furthermore, deletion of MoIst1 led to an increase in autophagy.

## Materials and Methods

### Strains, Culture Conditions, and Quantitative Real-Time PCR

The wild-type strain used in this research was Guy11. All strains were cultured on complete medium (CM), which was placed in an incubator at a temperature of 25°C and light for 16 h and darkness for 8 h alternately for 8 days. For different stress tests, 10 mM hydrogen peroxide (H_2_O_2_) and 0.8 mM paraquat, 0.004% sodium dodecyl sulfate (SDS), 30 μg/ml calcofluor white (CFW), 600 μg/ml Congo red (CR), 0.5 M NaCl, 0.5 M KCl, and 1 M sorbitol were added separately to CM. The PCR primers used in this research are listed in Supplementary Table 1.

### Targeted Gene Deletion and Complementation of Deletion Mutants

A high-throughput target-gene deletion strategy designed by Professor Jian-Ping Lu was used to knock out *MoIST1* in wild-type Guy11, and a copy of the native gene was inserted into Δ*Moist1* to obtain the complemented strain (Lu et al., 2014). The vector used for knockout and complementation was pKO3A, which was digested by *Hin*dIII/*Sail*I and pKD3, which were digested by *Eco*RI/*Bam*HI. The hygromycin resistance gene (*HPH*) and the bacterial bialophos resistance gene (*BAR*) were ligated with pKO3A and pKD3 by Clonexpress Multis One Step Cloning Kit and ClonExpress II One Step Cloning Kit (Vazyme, China), respectively, as selection markers. *Agrobacterium tumefaciens*-mediated transformation (ATMT) was used to transform the vector into WT or Δ*Moist1* to obtain Δ*Moist1* or *Moist1C*, respectively. Successful replacement was first verified by PCR, and then the copy number was determined by quantitative real-time PCR (qPCR). The PCR primers used in this study are listed in Supplementary Table 1.

### Phenotypic Characterization

For sporulation, 3 ml of double-distilled water (ddH_2_O) was added to the CM plate to obtain conidia, and then quantitative statistics were performed. Appressorium development was observed on hydrophobic surfaces with 20 μl of spore suspensions (5 × 10^4^ conidia/ml) at 22°C. Conidial germination was observed at 4 h. Appressorium formation was observed at 4, 6, 8, and 24 h. Collapse of appressoria was observed at 24 h, and the glycerol solutions used were 0.25, 0.5, and 1.0 M. Lipid droplet staining was observed at 0, 8, 16, and 24 h using the fluorescent dye boron dipyrromethene (BODIPY) (Thermo Fisher Scientific, United States), and tricyclazole was used in spore suspension to inhibit the formation of melanin in the appressorium. Glycogen staining was observed at 0, 8, 16, and 24 h using KI/I_2_ solution.

### Pathogenicity Assay

Fourteen-day-old rice seedlings (*Oryza sativa* cv CO-39) and 8-day-old barley were used. Mycelial plugs of WT, Δ*Moist1* and *Moist1C* were inoculated on detached barley, which was placed in an incubator with a temperature of 25°C and light for 16 h and darkness for 8 h alternately for 4 days. Spore suspensions (5 × 10^4^ conidia/ml) were sprayed onto detached barley leaves, which were placed in an incubator with a temperature of 25°C and light for 16 h and darkness for 8 h alternately for 4 days. Spore suspensions (1 × 10^5^ conidia/ml) and 0.4% (w/v) gelatin were mixed in equal volumes, sprayed onto rice seedlings and cultured at 22°C with darkness for 2 days and 25°C and then with light for 16 h and darkness for 8 h alternately for 4 days. Infection assays were performed on detached barley with spore suspensions (5 × 10^4^ conidia/ml) and cultured for 48 h and 72 h after inoculation. Then, the leaves were decolorized with methanol and stored in lactophenol (Kim et al., 2005).

### Western Blot Analysis

Mycelia collected from CM plates were cultured in liquid CM at 138 rpm at 28°C for 48 h and then shifted to nitrogen starvation (SD-N) medium at 180 rpm at 28°C for 2 h and 4 h to induce autophagy. The location of GFP-MoAtg8 under CM and SD-N conditions was analyzed using a microscope (Eclipse 80i 1003 OI). For testing phosphorylated Pmk1, mycelia collected from CM plates were cultured in liquid YEG at 138 rpm at 28°C for 48 h (Qu et al., 2021). The primary antibodies used in this research were GFP (GFP-Tag Rabbit mAb, Huabio, Hangzhou, China), phosphorylated Pmk1 (Cell Signaling Technology, Inc), non-phosphorylated Pmk1 (Santa Cruz Biotechnology, Inc), phosphorylated Osm1 (Cell Signaling Technology, Inc), and GAPDH (Huabio, Hangzhou, China). The secondary antibody was goat anti-rabbit/mouse IgG HRP (Beyotime, Shanghai, China).

## Results

### MoIst1 Is Involved in Sporulation and Appressorium Development in *Magnaporthe oryzae*

**FIGURE 1:**
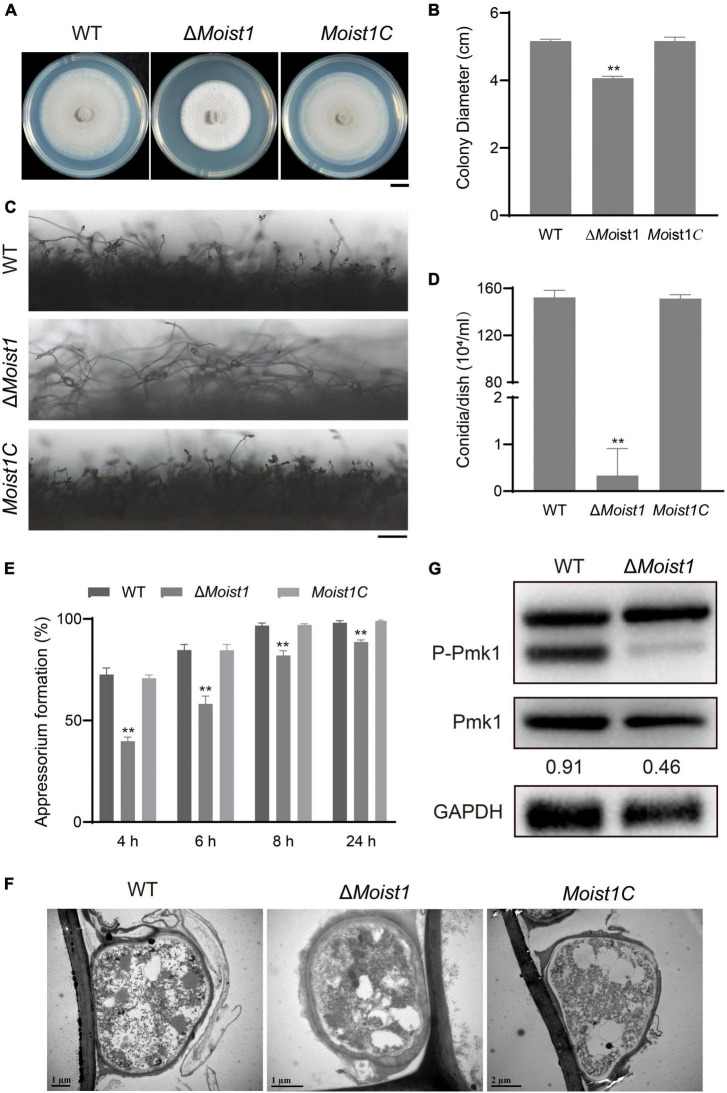
MoIst1 is involved in sporulation and appressorium development in *M. oryzae*. **(A)** WT, Δ*Moist1* and *Moist1C* were cultured on CM plates for 8 days to observe colony morphology. Bar = 1 cm. **(B)** Colony diameters of WT, Δ*Moist1*, and *Moist1C*. The standard deviation is represented by error bars. Tukey’s test was used to test significant differences: **P < 0.01. **(C)** Conidiophores of WT, Δ*Moist1*, and *Moist1C*. Bar = 100 μm. **(D)** Conidiation of WT, Δ*Moist1* and *Moist1C*. The standard deviation is represented by error bars. Tukey’s test was used to test significant differences: **P < 0.01. **(E)** Appressorium formation rates of WT, Δ*Moist1*, and *Moist1C* on hydrophobic surfaces. The standard deviation is represented by error bars. Tukey’s test was used to test significant differences: **P < 0.01. **(F)** Appressoria of WT, Δ*Moist1*, and *Moist1C* were observed by transmission electron microscopy (TEM). **(G)** Non-phosphorylated and phosphorylated Pmk1 levels were detected by anti-ERK1/ERK2 MAPK and anti-phospho-p44/42 MAPK antibodies, respectively. GAPDH was used as an internal reference for protein standardization by Western blot.

Previously, we identified ESCRT-0 complex, and showed that its two components Δ*Movps27* and Δ*Mohse1* participated in fungal development, pathogenicity, autophagy, and ER-phagy in *M. oryzae* (Sun et al., 2021). In this study, we focused on MoIst1, a subunit of ESCRT III to explore its function in *M. oryzae*. Sequence alignment analysis showed that MGG_01765 shared 34.49% identity with *S. cerevisiae* Ist1 (Supplementary Figure 1A), and it was renamed according to the ortholog in *S. cerevisiae* called MoIst1. A high-throughput target-gene deletion strategy designed by Lu et al. (2007) was used to knock out *MoIST1* in wild-type Guy11, and a copy of the native gene was inserted into Δ*Moist1* to obtain the complemented strain (Supplementary Figures 1B,C). To explore the biological functions of *MoIST1*, we first focused on analyzing the differences from WT in terms of vegetative growth, sporulation and appressorium development. The mycelial growth of Δ*Moist1* was reduced by approximately 25% compared with the growth of WT and *Moist1C* (Figures 1A,B). Compared to many conidiophores bearing conidia in WT and *Moist1C*, the conidiophores of Δ*Moist1* had almost no conidia (Figure 1C), and as a result, Δ*Moist1* produced 0.0022 ± 0.0038 × 10^4^ conidia/cm^2^, far less than WT (0.9901 ± 0.0392 × 10^4^ conidia/cm^2^) and *Moist1C* (0.9836 ± 0.0209 × 10^4^ conidia/cm^2^) (Figure 1D). Although sporulation decreased significantly, spore germination was not affected (Supplementary Figure 1D). Obviously different from the unaffected germination, in Δ*Moist1*, the formation speed and rate of appressoria were significantly reduced, and the appressorial formation rate was only approximately 88% at 24 h, indicating the delayed appressorial formation in Δ*Moist1* (Figure 1E). In the process of statistics of the formation rate of appressoria, we found that there were great differences in the appearance of appressoria between WT, Δ*Moist1* and *Moist1C.* To clearly distinguish the differences between them, we observed the appressoria by transmission electron microscopy (TEM), and as shown in Figure 1F, compared with WT and *Moist1C*, the cell wall of the appressoria of Δ*Moist1* is disrupted and unstable, showing obvious mucilage layer.

As the Pmk1 MAPK cascade is a signaling pathway that controls appressorium formation and Δ*Mopmk1* fails to form appressoria (Xu and Hamer, 1996), we detected the levels of non-phosphorylated and phosphorylated Pmk1 proteins in WT and Δ*Moist1*. As shown in Figure 1G, the protein levels of non-phosphorylated Pmk1 in Δ*Moist1* showed no significant difference from WT, while the level of phosphorylated Pmk1 showed a significant decline. Taken together, MoIst1 is important for vegetative growth, sporulation and appressorium development in *M. oryzae*.

### MoIst1 Is Required for Plant Penetration and Pathogenicity in *Magnaporthe oryzae*

**FIGURE 2:**
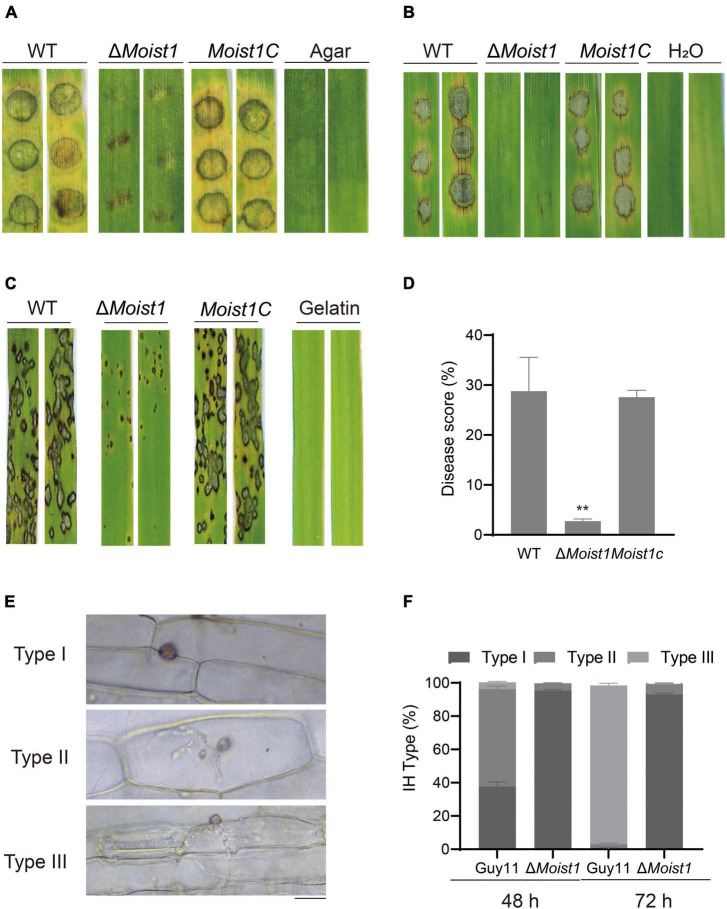
MoIst1 is required for plant penetration and pathogenicity in *M. oryzae*. **(A)** Pathogenicity of mycelial plugs on barley leaves. **(B)** Pathogenicity of spore suspensions (5 × 10^4^ ml^–1^) on barley leaves. **(C)** Pathogenicity of spore suspensions (5 × 10^4^ ml^–1^) on rice leaves. **(D)** Five-centimeter-long leaves of each rice leaf were taken for statistics of the number of disease spots. The standard deviation is represented by error bars. Tukey’s test was used to test significant differences: **P < 0.01. **(E)** The IHs of WT and Δ*Moist1* were divided into three types. Bar = 20 μm. **(F)** The three types of IHs were quantified and statistically significant at 48 and 72 hpi. The standard deviation is represented by error bars. Tukey’s test was used to test significant differences: **P < 0.01.

To further examine the functions of MoIst1 in pathogenesis, assays were performed on barley and rice. Mycelial plugs were inoculated on detached barley leaves, and severe lesions were observed on leaves inoculated with WT and *Moist1C* (Figure 2A). However, small and restricted lesions were observed on leaves inoculated with Δ*Moist1*. Similar results were obtained on barley leaves and rice seedlings sprayed with conidial suspensions (5 × 10^4^ conidia/ml) (Figures 2B,C). The disease score of Δ*Moist1* on rice seedlings was 2.75 ± 0.26%, which was significantly lower than those of WT and *Moist1C*, which were 28.78 ± 3.91% and 27.58 ± 0.78%, respectively (*P* < 0.01) (Figure 2D).

For further exploration of the pathogenicity reduction in Δ*Moist1*, penetration assays were performed using detached barley leaves. Invasive hyphae (IHs) for all strains were classified into 3 types (Type I, IHs with no hyphal penetration; Type II, IH branches in only one cell; Type III, IH branches in more than one cell) (Figure 2E). Various types of IH were quantified and statistically analyzed. At 48 h, in WT, nearly 60% of IHs were type II; in contrast, only 4% of IHs were type II in Δ*Moist1*. At 72 h, nearly 95% of IHs were type III, while few were observed in Δ*Moist1*, and mostly type I IHs were still present (Figure 2F). Extending slowly to neighboring cells of Δ*Moist1* suggested that MoIst1 is important for appressorium-mediated infection, invasive hyphal growth and pathogenicity.

### MoIst1 Is Essential for Appressorium Turgor and the Degradation of Glycogen and Lipid Droplets

**FIGURE 3:**
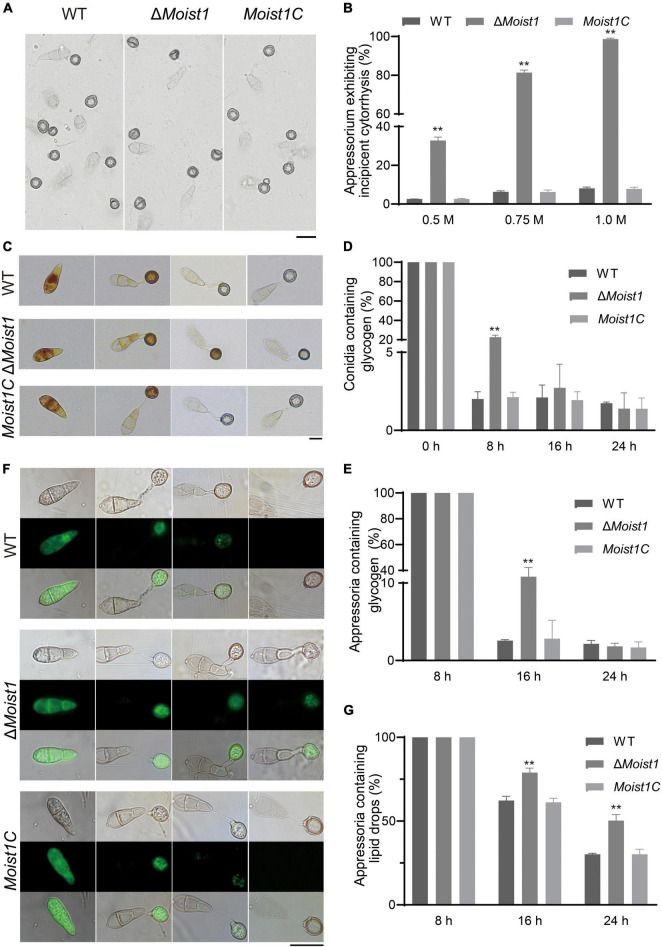
MoIst1 is essential for appressorium turgor and the degradation of glycogen and lipid droplets. **(A)** Collapse of appressoria at 1.0 M glycerol. Bar = 20 μm. **(B)** Collapse rates of appressoria exposed to 0.25, 0.5, and 1.0 M glycerol solutions. The standard deviation is represented by error bars. Tukey’s test was used to test significant differences: **P < 0.01. **(C)** Changes in glycogen during appressorium development. Bar = 20 μm. **(D)** The proportion of conidia containing glycogen. The standard deviation is represented by error bars. Tukey’s test was used to test significant differences: **P < 0.01. **(E)** The proportion of appressoria containing glycogen. The standard deviation is represented by error bars. Tukey’s test was used to test significant differences: **P < 0.01. **(F)** Changes in lipid droplets during appressorium development. Bar = 50 μm. **(G)** The proportion of appressoria containing lipid droplets. The standard deviation is represented by error bars. Tukey’s test was used to test significant differences: **P < 0.01.

Appressoria produce a turgor up of to 8.0 MPa by accumulating high concentrations of glycerol and other polyols to rupture the leaf epidermis (deJong et al., 1997). Considering that the formation of appressoria was delayed in Δ*Moist1* but that the formation rate tended to 90% after 24 h, we hypothesized that slow infection may be associated with turgor pressure. Appressoria were exposed to different concentrations of glycerol, and their collapse rate indicated the level of turgor (Howard et al., 1991; Lu et al., 2007). As shown in Figures 3A,B, the collapse rates of Δ*Moist1* were significantly higher than those of WT and *Moist1C*. At a glycerol concentration of 1.0 M, almost 100% of the appressoria of Δ*Moist1* collapsed, while only approximately 8% of the appressoria WT and *Moist1C* collapsed.

In *M. oryzae*, the accumulation of appressorium turgor requires autophagy to degrade the lipids and glycogen in conidia and is then transported to the appressoria through the germ tube to increase the appressorium turgor (deJong et al., 1997; Foster et al., 2003; Wilson and Talbot, 2009). In view of this, the translocation and degradation of lipid droplets and glycogen were examined at 0, 8, 16, and 24 h, respectively. As shown in Figures 3C–E, compared with WT and *Moist1C*, the translocation and degradation of glycogen in Δ*Moist1* were delayed to a certain extent. Glycogen of WT and *Moist1C* could be completely transported from spores to appressoria at 8 h, and the transport of Δ*Moist1* was completed at 16 h. Glycogen of WT and *Moist1C* could be degraded at 16 h in appressoria, and the degradation of Δ*Moist1* was completed at 24 h. Unlike glycogen, there is no difference between WT, Δ*Moist1* and *Moist1C* in the translocation of lipid droplets, while their degradation was delayed in Δ*Moist1* (Figures 3F,G). The above results indicate that MoIst1 is essential for appressorium turgor and the degradation of glycogen and lipid droplets.

### MoIst1 Affects Cell Wall Integrity

**FIGURE 4:**
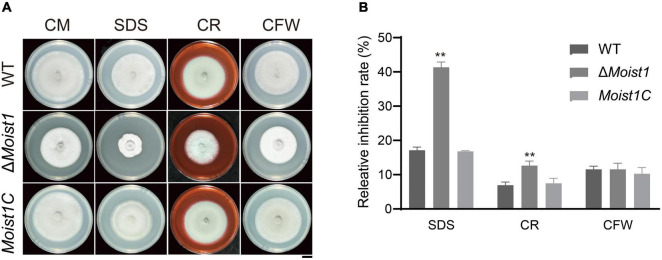
Deletion of MoIst1 increases sensitivity to cell wall integrity stress. **(A)** SDS (0.005%), CFW (30 μg/ml), and CR (600 μg/ml) were added separately to CM to culture WT, Δ*Moist1*, and *Moist1C*. Bar = 1 cm. **(B)** Relative inhibition rates of WT, Δ*Moist1*, and *Moist1C* on CM with SDS (0.004%), CFW (30 μg/ml), and CR (600 μg/ml). The standard deviation is represented by error bars. Tukey’s test was used to test significant differences: **P < 0.01.

The cell wall integrity (CWI) signaling pathway is one of the key mechanisms by which fungi resist external stress and maintain cell survival (Levin, 2005; Li et al., 2012). To explore the biological functions of *MoIST1* in response to cell wall integrity stress, WT, Δ*Moist1*, and *Moist1C* were incubated on CM with 0.004% sodium dodecyl sulfate (SDS), 600 μg/ml Congo red (CR) and 30 μg/ml calcofluor white (CFW). As shown in Figures 4A,B, Δ*Moist1* was more sensitive to SDS and CR than WT and *Moist1C*. This result suggested that disruption of MoIst1 results in increased sensitivity to cell wall integrity stress.

### Deletion of MoIst1 Increases Sensitivity to Oxidative Stress

**FIGURE 5:**
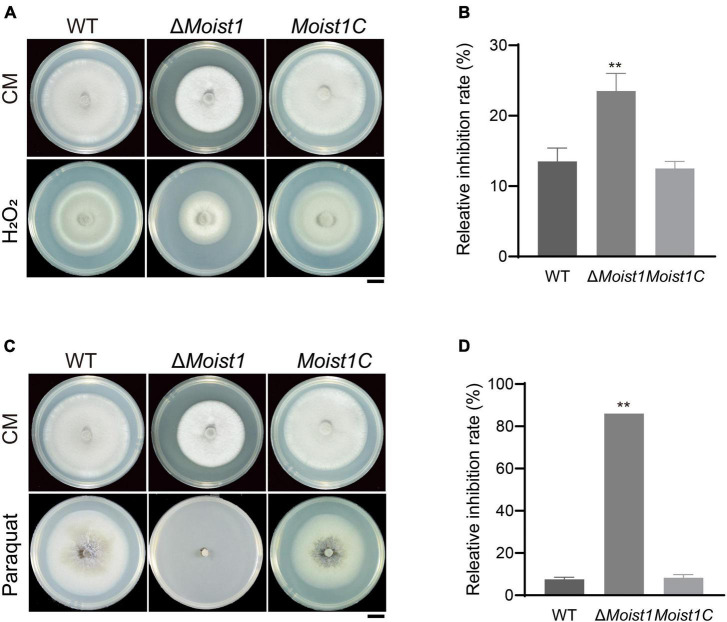
Deletion of MoIst1 increases sensitivity to oxidative stress. **(A)** Hydrogen peroxide (H_2_O_2_, 10 mM) was added separately to CM to culture WT, Δ*Moist1*, and *Moist1C*. Bar = 1 cm. **(B)** Relative inhibition rates of WT, Δ*Moist1* and *Moist1C* on CM with 10 mM hydrogen peroxide (H_2_O_2_). The standard deviation is represented by error bars. Tukey’s test was used to test significant differences: **P < 0.01. **(C)** Paraquat (0.8 mM) was added separately to CM to culture WT, Δ*Moist1*, and *Moist1C*. Bar = 1 cm. **(D)** Relative inhibition rates of WT, Δ*Moist1*, and *Moist1C* on CM with 0.8 mM paraquat. The standard deviation is represented by error bars. Tukey’s test was used to test significant differences: **P < 0.01.

Reactive oxygen species (ROS) are secreted by plants to prevent the invasion of pathogens (Li et al., 2012). Although the turgor of Δ*Moist1* is significantly lower than that of WT, there are still some mutants that can successfully achieve invasion, though the invasion speed is significantly slower than that of WT. Therefore, we hypothesize that inability to scavenge ROS is the reason that Δ*Moist1* IH fails to colonize adjacent cells. To confirm this, the radial growth rates of WT, Δ*Moist1* and *Moist1C* on CM containing the oxidative stress agent hydrogen peroxide (H_2_O_2_) and paraquat were examined. As shown in Figures 5A–D, Δ*Moist1* was more sensitive to H_2_O_2_ and paraquat, especially on plates containing paraquat, and Δ*Moist1* hardly grew on them. Our findings suggested that disruption of MoIst1 results in increased sensitivity to oxidative stress.

### MoIst1 Is Involved in Responses to Hyperosmotic Stresses

**FIGURE 6:**
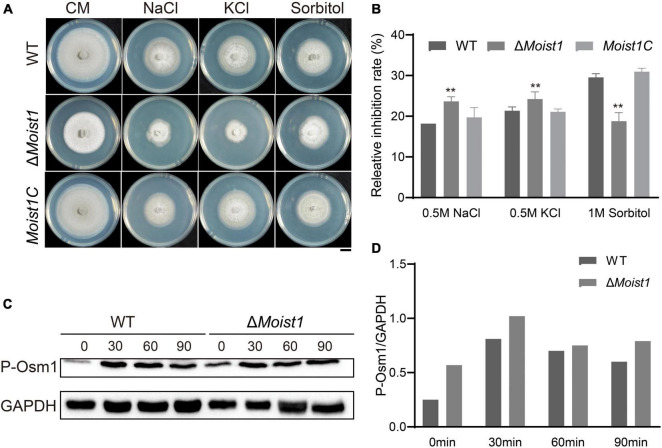
Deletion of MoIst1 increases sensitivity to hyperosmotic stress. **(A)** 0.5 M NaCl, 0.5 M KCl, and 1 M sorbitol were added separately to CM to culture WT, Δ*Moist1* and *Moist1C*. Bar = 1 cm. **(B)** Relative inhibition rates of WT, Δ*Moist1*, and *Moist1C* on CM with 0.5 M NaCl, 0.5 M KCl and 1 M Sorbitol. The standard deviation is represented by error bars. Tukey’s test was used to test significant differences: **P < 0.01. **(C)** Phosphorylated Osm1 levels were detected. GAPDH was used as an internal reference for protein standardization by Western blot. **(D)** The content of phosphorylated Osm1 relative to GAPDH at different time points in WT and Δ*Moist1*.

The HOG1 pathway is a signaling pathway widely present in fungi, and under hypertonic conditions, activating HOG1 to promote the expression of genes related to glycerol synthesis is essential for fungal growth (Maeda et al., 1994; Akhtar et al., 1997; Ferrigno et al., 1998; Hohmann, 2002; Saito and Tatebayashi, 2004; Noguchi et al., 2007; Zheng et al., 2012). To test the sensitivity of Δ*Moist1* to hyperosmotic stress, WT, Δ*Moist1* and *Moist1C* were incubated on CM with 0.5 M NaCl, 0.5 M KCl and 1 M Sorbitol. As shown in Figures 6A,B, the loss of MoIst1 resulted in increased sensitivity to ionic hypertonic stresses, indicating that MoIst1 played important roles in adapting to hypertonic stresses.

In *M. oryzae*, MAPK Osm1 mediates the hyperosmotic stress response (Li et al., 2012). To verify whether MoIst1 regulates this pathway by affecting the phosphorylation of Osm1, the phosphorylation levels of Osm1 were monitored in WT and Δ*Moist1* treated with 0.5 M NaCl. As shown in Figures 6C,D, when treated with 0.5 M NaCl, the phosphorylation level of Osm1 was increased in WT and Δ*Moist1* in the first 30 min and then decreased later. It is worth noting that the phosphorylation levels of Osm1 in Δ*Moist1* were always higher than those in WT at all times, indicating that MoIst1 is involved in controlling the phosphorylation of the Osm1 kinase to adapt to hyperosmotic stress.

### MoIst1 Is Required for Autophagy Process

Autophagy, a conserved intracellular degradation system, in which some damaged proteins or organelles are encapsulated by double-membrane autophagic vesicles and then delivered to lysosomes or vacuoles is degraded and recycled (Zhu et al., 2019). ESCRT is involved in the formation of a variety of neurodegenerative diseases by inhibiting autophagy degradation, indicating that ESCRT plays an important role in the regulation of autophagy (Filimonenko et al., 2007; Krasniak and Ahmad, 2016; Wang et al., 2019). Accordingly, we wondered whether MoIst1 is also involved in the regulation of autophagy.

**FIGURE 7:**
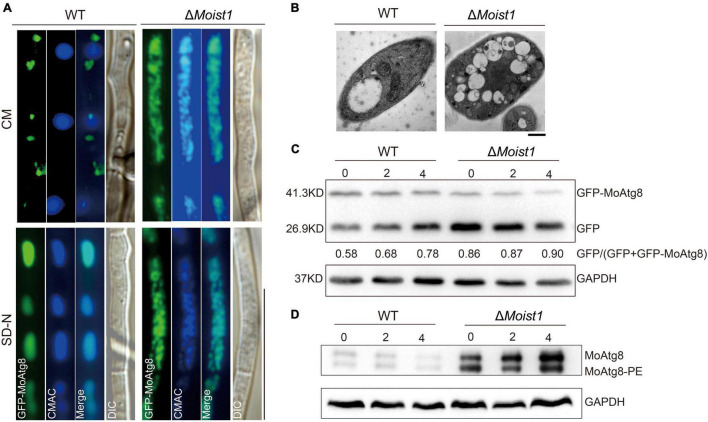
MoIst1 is required for autophagy process. **(A)** Localization of GFP-MoAtg8 in WT and Δ*Moist1*. Strains were grown in liquid CM medium at 28°C for 48 h and then shifted to liquid SD-N medium for 4 h. Mycelia were stained with CMAC and examined under a fluorescence microscope. Bar = 50 μm. **(B)** WT and Δ*Moist1* were cultured on solid CM for 7–9 days and then in CM liquid medium cultured at 28°C for 48 h continuously shaken at 138 rpm. Bar = 0.5 μm. **(C)** Degradation of GFP-MoAtg8 was detected in WT and Δ*Moist1* under nitrogen starvation for 0, 2, and 4 h by GFP antibodies. GAPDH was used as an internal reference for protein standardization by Western blot. **(D)** The conversion of MoAtg8 to Lipidation of MoAtg8, MoAtg8-PE, was compared in WT and Δ*Moist1* with anti-Atg8 antibody. GAPDH was used as an internal reference for protein standardization by Western blot.

A GFP-MoAtg8 fusion protein was used in this research to detect whether there is a link between MoIst1 and autophagy. First, the subcellular localization of GFP-MoAtg8 was observed. As shown in Figure 7A, GFP-MoAtg8 was positioned around the vacuole in the form of dots in WT, while in Δ*Moist1*, it was observed that the entire vacuole was uniformly green without obvious localization under CM conditions. After 4 h of nitrogen starvation, the entire vacuole was uniformly green without obvious localization in WT. In Δ*Moist1*, there was no difference from the results observed under CM conditions. It is worth noting that vacuoles in Δ*Moist1* seemed different from WT. For further confirmation, transmission electron microscopy (TEM) was used. As shown in Figure 7B, vacuoles in Δ*Moist1* were irregular and fragmented, which is different from the complete central large vacuole of WT.

To further verify the connection between MoIst1 and autophagy, Western blotting was performed to detect the content of full-length GFP-MoAtg8 and free GFP in WT and Δ*Moist1*. As shown in Figure 7C, autophagic flux of Δ*Moist1* was higher than WT.

As reported, the absence or dysfunction of ESCRT-III affects phagophore closure and the fusion of autophagosomes with lysosomes, resulting in abnormal autophagy (Lee et al., 2007; Rusten et al., 2007). Therefore, we detected endogenous Atg8/Atg8-phosphatidylethanolamine (Atg8-PE) in WT and Δ*Moist1*. As shown in Figure 7D, we founded in that Δ*Moist1* the conversion of MoAtg8 to MoAtg8-PE was not affected,.

## Discussion

Endosomal sorting complexes required for transport (ESCRT) are a kind of protein complex that can identify and sort ubiquitinated proteins and consist of ESCRT-0, ESCRT-I, ESCRT-II, ESCRT-III, and some auxiliary complexes. ESCRT-III, which contains IST1, is essential for the biogenesis of MVBs, budding, and abscission during cytokinesis (Raiborg and Stenmark, 2009). In this research, we identified that MoIst1 is involved in sporulation, appressorium development, plant penetration, fungal pathogenicity, and autophagy in *M. oryzae*.

Appressoria produced by the conidial germination of *M. oryzae* can destroy the leaf epidermis by accumulating high concentrations of glycerin and other polyols to generate a turgor of up to 8.0 MPa (Fernandez and Orth, 2018). Our research showed that Δ*Moist1* showed not only a significant decrease in the number of sporulation compared with WT (Figure 1B) but also that the infection ability of appressoria was slowed down and weakened due to the obvious reduction in turgor (Figures 2F, 3B). Research in *M. oryzae* has shown that under the action of the *PMK1* MAPK pathway, the compartmentalization and rapid degradation of glycogen and lipid drops cause the generation of turgor (Thines et al., 2000). In our study, the degradation of lipid droplets and glycogen was delayed in Δ*Moist1* (Figures 3E,G), and the phosphorylated Pmk1 was also lower (Figure 1H) showed that in Δ*Moist1*, the lower phosphorylated Pmk1 is one reason for the delayed degradation of lipid droplets and glycogen. In addition, autophagy has also been shown to be involved in the degradation of lipid droplets and glycogen (Liu et al., 2007), and our results in Figure 7C show the increased autophagic flux in Δ*Moist1*, suggesting that dysregulated autophagy also participates in the control of turgor by influencing the degradation of lipid droplets and glycogen.

The CWI signaling pathway is one of the key mechanisms by which fungi resist external stress and maintain cell survival (Levin, 2005; Li et al., 2012). As shown in Figure 4A, Δ*Moist1* was more sensitive to cell wall stress than WT. At the same time, the results of TEM in Figure 1F also showed that the cell wall of Δ*Moist1* had an obvious mucilage layer. Combined with the results in Figure 4, it showed that the cell wall of Δ*Moist1* was damaged and unstable. In addition, Δ*Moist1* was sensitive to oxidative stress. Taken together, MoIst1 participates in the maintenance of external stresses by maintaining cell wall integrity and resisting oxidative stress.

Autophagy is an essential, conserved self-eating process that cells perform to allow degradation of intracellular components. The complete process of autophagy consists of three steps: induction, autophagosome formation, and autophagosome-lysosome fusion and degradation (Mizushima et al., 2008; Nixon, 2013). Studies in *Drosophila*, *Arabidopsis*, yeast, mice, and mammals show that inhibiting the expression of ESCRT affects the number and structure of autophagosomes, resulting in a problem in autophagosome-lysosome fusion, indicating that ESCRT plays an important role in the regulation of autophagy (Lloyd et al., 2002; Spitzer et al., 2015; Feng et al., 2020; Schafer and Schuck, 2020). MAPT protein (the hallmark pathology in Alzheimer’s disease) aggregation inhibits the expression of IST1 through the CEBPB-ANP32A-INHAT pathway, which hinders the formation of the ESCRT-III complex, inhibits the fusion of autophagosomes and lysosomes, and leads to autophagy disorder (Feng et al., 2020). In *Arabidopsis thaliana*, the final stage of autophagosome formation is the closure of the double-layer membrane, and deletion of CHMP1 (a subunit of ESCRT-III) causes autophagosomes to not close, which results in the appearance of problematic autophagosomes (Spitzer et al., 2015).

In our study, we found that compared with WT, the deletion of MoIst1 triggered quick lipidation of MoAtg8 and degradation of the autophagic marker protein GFP-MoAtg8 under nitrogen starvation conditions, indicating that the deletion of MoIst1 triggered quick lipidation of the conversion of MoAtg8 and the autophagic flux. Phenotypic characterization of Δ*Moist1* also revealed defects in reduced turgor pressure, delayed translocation and degradation of glycogen and lipids, and reduced pathogenicity, similar to ATG-deficient mutants (Veneault-Fourrey et al., 2006; Liu et al., 2007). Although our results showed that MoIst1 is involved in autophagy, it is different from studies that deletion of CHMP1 (a subunit of ESCRT-III) resulted in autophagosomes to not close (Spitzer et al., 2015), or inhibition of IST1 expression by MAPT protein aggregation inhibited the fusion of autophagosomes and lysosomes (Feng et al., 2020), and so far, our existing evidence cannot prove that MoIst1 affected the fusion of autophagosome and vacuole, but only in Δ*Moist1* autophagy was enhanced. However, the specific mechanism is still unclear, and further research is needed. In conclusion, we found that MoIst1 is essential for vegetative growth, sporulation, appressorium development, response to oxidative stress, cell wall stress, hyperosmotic stress, and autophagy. And as for other changes in phenotypic characterization of Δ*Moist1* (including defects in reduced turgor pressure, delayed translocation and degradation of glycogen and lipids, and reduced pathogenicity) is caused by the enhanced autophagy.

## Data Availability Statement

The original contributions presented in the study are included in the article/Supplementary Material, further inquiries can be directed to the corresponding author.

## Author Contributions

LS and XL contributed to experimental design. LS, HQ, and WZ contributed to experiments. LS, HQ, MW, WZ, ML, YW, XZ, and LL contributed to data analysis and scripts. XL, FL, and, JL supplied experimental conditions. LS, XL, FL, and JL wrote the manuscript. All authors contributed to the article and approved the submitted version.

## Conflict of Interest

The authors declare that the research was conducted in the absence of any commercial or financial relationships that could be construed as a potential conflict of interest.

## Publisher’s Note

All claims expressed in this article are solely those of the authors and do not necessarily represent those of their affiliated organizations, or those of the publisher, the editors and the reviewers. Any product that may be evaluated in this article, or claim that may be made by its manufacturer, is not guaranteed or endorsed by the publisher.

## Supplementary Material

Supplementary Figure 1 **(A)** Multiple sequence alignment of MoIst1 homolog proteins using CLC Main Workbench. **(B)** Successful replacement was verified by PCR. **(C)** The copy number was determined by quantitative real-time PCR (qPCR). **(D)** Conidial germination rates of WT, Δ*Moist1*, and *Moist1C* at 4 h. The standard deviation is represented by error bars. Tukey’s test was used to test significant differences: ^**^*P* < 0.01, **P* < 0.05.
