# Supplementary material for: A Subunit of ESCRT-III, MoIst1, Is Involved in Fungal Development, Pathogenicity, and Autophagy in Magnaporthe oryzae
Source: Front Plant Sci. 2022 Apr 7;13:845139. doi: 10.3389/fpls.2022.845139 (PMC9021896; doi:10.3389/fpls.2022.845139)
Supplement: Supplementary file 1 [file Table_1.docx]

**Table S1** Primers used in this study

| **Primer name** | **Primer** |
| --- | --- |
| **Primers for gene knock out** | |
| MoIst1-UpF | AGGCTAACTGACACTCTAGA CCTGAATGCCTCTTGGAC |
| MoIst1-UpR | CATTCATTGTTGACCTCCACTA TTCGGATGTTGTAGTGGTTA |
| MoIst1-DownF | GGGCAAAGGAATAGAGTAGATGTTCTATGTAGGCATCTAGTCAG |
| MOIst1-DownR | CGACGGCCAGTGCCAAGCTT AGGCTTAGCAGAGTCGTAT |
| MoIst1-Long-F | CTGGAGAATCAAGTGCTAGA |
| MoIst1-SF | AAGTTCGGCAAGGAGTTC |
| MoIst1-SR | CGGCATCTACCATCTTCTT |
| YHPH-R | GATAATAATGTCCTCGTTCC |
| HPH-F | TAGTGGAGGTCAACAATGAATG |
| HPH-R | CATCTACTCTATTCCTTTGCC |
| MoIst1C-F | TCACAATCACTAGTGAATTCCTGGAGAATCAAGTGCTAGA |
| MoIST1C-R | ACCATCCCGGGGATGGATCC TCTTTTCAATGCGGCAAAC |
| **Primers for quantitative real-time PCR** | |
| RT-Tubulin-F | ACAACTTCGTCTTCGGTCAG |
| RT-Tubulin-R | GTGATCTGGAAACCCTGGAG |
| RT-HPH-F | ATGTCCTGCGGGTAAATAGC |
| RT-HPH-R | GATGCAATAGGTCAGGCTCTC |
