# Supplementary figures and images for: A Subunit of ESCRT-III, MoIst1, Is Involved in Fungal Development, Pathogenicity, and Autophagy in Magnaporthe oryzae
Source: Front Plant Sci. 2022 Apr 7;13:845139. doi: 10.3389/fpls.2022.845139 (PMC9021896; doi:10.3389/fpls.2022.845139)

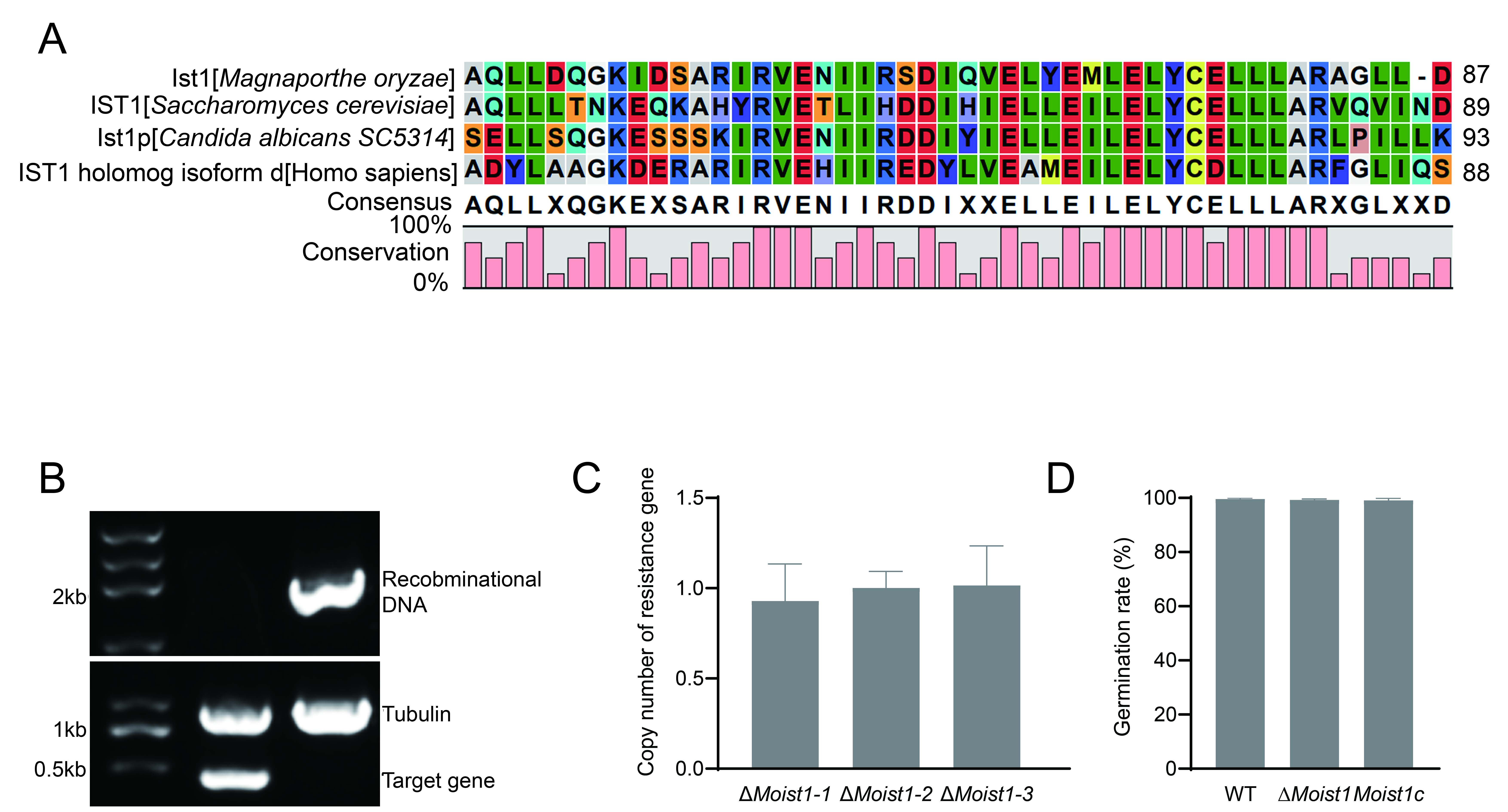

Supplement: Supplementary Figure 1 — (A) Multiple sequence alignment of MoIst1 homolog proteins using CLC Main Workbench. (B) Successful replacement was verified by PCR. (C) The copy number was determined by quantitative real-time PCR (qPCR). (D) Conidial germination rates of WT, ΔMoist1, and Moist1C at 4 h. The standard deviation is represented by error bars. Tukey’s test was used to test significant differences: **P < 0.01, *P < 0.05. [file Image_1.JPEG]
